# Modeling the Mechanics of Cancer: Effect of Changes in Cellular and Extra-Cellular Mechanical Properties

**DOI:** 10.3389/fonc.2013.00145

**Published:** 2013-06-11

**Authors:** Parag Katira, Roger T. Bonnecaze, Muhammad H. Zaman

**Affiliations:** ^1^McKetta Department of Chemical Engineering, The University of Texas at Austin, Austin, TX, USA; ^2^Department of Biomedical Engineering, Boston University, Boston, MA, USA

**Keywords:** cancer modeling, mechanical forces, cell-material interactions, cell–cell interaction, review

## Abstract

Malignant transformation, though primarily driven by genetic mutations in cells, is also accompanied by specific changes in cellular and extra-cellular mechanical properties such as stiffness and adhesivity. As the transformed cells grow into tumors, they interact with their surroundings via physical contacts and the application of forces. These forces can lead to changes in the mechanical regulation of cell fate based on the mechanical properties of the cells and their surrounding environment. A comprehensive understanding of cancer progression requires the study of how specific changes in mechanical properties influences collective cell behavior during tumor growth and metastasis. Here we review some key results from computational models describing the effect of changes in cellular and extra-cellular mechanical properties and identify mechanistic pathways for cancer progression that can be targeted for the prediction, treatment, and prevention of cancer.

## Introduction

**Figure 1:**
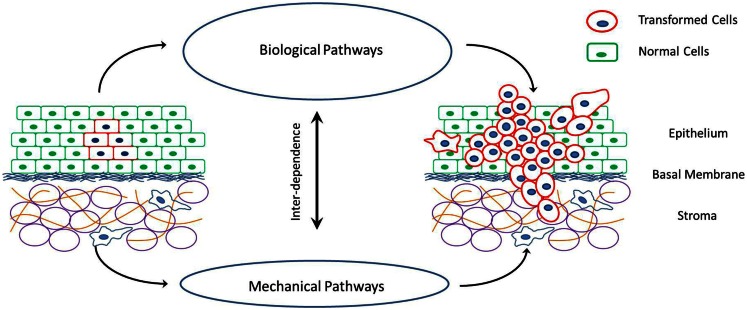
**Schematic of cancer progression in a tissue, and the interplay between the mechanical and biological factors that drive these processes of cell proliferation, invasion of surrounding tissue and metastasis via individual or collective cell migration**.

Cancer is a disease rooted in the dis-regulation of cellular signaling pathways that control cell proliferation and apoptosis. This is generally caused by mutations in genes that express key proteins involved in these biochemical reactions. However, cancer is also accompanied by specific changes in the mechanical properties of cells and their surrounding extra-cellular environment (Figure 1). For example, cancerous cells are less stiff compared to their healthy counter parts (Suresh, 2007). This decrease in cell stiffness with malignant transformation has been observed in a variety of cancers such as breast cancer, lung cancer, renal cancer, prostate cancer, oral cancer, skin cancer, and so on (Guck et al., 2005; Cross et al., 2007; Suresh, 2007; Remmerbach et al., 2009; Fuhrmann et al., 2011; Jonas et al., 2011; Plodinec et al., 2012). Furthermore, the decrease in cell stiffness seems to be greater in cells with higher malignancy and metastatic potential (Swaminathan et al., 2011). Cancerous cells also have increased acto-myosin cortex contractility as compared to corresponding healthy cells (Jonas et al., 2011; Kraning-Rush et al., 2012). This has been observed in response to the stretching of cells by external stimuli. Apart from the cortex stiffness and contractility, cancerous cells also undergo changes in their ability to physically bind to their neighbors and the surrounding extra-cellular elements at different stages of cancer progression (Paredes et al., 2005; Ribeiro et al., 2010a,b). This is caused by the up or down regulation of specific adhesion proteins on the cell surface and affect the growth rate, shape, and invasiveness of tumors. Accompanying the changes in cellular mechanical properties are also some very specific changes in the mechanical properties of the extra-cellular environment. Tumors with high invasive potentials have a stiff extra-cellular environment (Erler and Weaver, 2009; Levental et al., 2009). The tumor extra-cellular environment consists primarily of fibrous tissue made up of collagen fibers. With malignant transformation of cells, an increase in the cross-linking of these fibers and a consequent stiffening of the extra-cellular matrix (ECM) environment has been observed (Erler et al., 2006). Once again this observation is common for a variety of cancers. Also, with the advent of metastasis and the invasion of the extra-cellular environment by cells of a growing tumor, the fibers in the ECM undergo extensive remodeling in terms of degradation, re-polymerization, and alignment (Alini and Losa, 1991; Vijayagopal et al., 1998; Zhang et al., 2003; Yang et al., 2004; Paszek et al., 2005; Vader et al., 2009). This realignment of ECM fibers and strain-induced stretching can alter the ECM mechanical properties as shown by Stein et al. (2011).

There is an increasing interest in the mechanics of cancer progression with an aim to identify mechanistic pathways that can be targeted for the prediction, treatment, and even prevention of cancer. With this in mind it is important to understand the effect of these peculiar changes in cellular and extra-cellular mechanical properties on tumor growth and metastatic potential. The potential influence of mechanical property changes on cell behavior during cancer progression has been discussed in recent commentaries and insight articles (Peyton et al., 2007; Kumar and Weaver, 2009; Fritsch et al., 2010). The underlying idea is that mechanical forces acting on cells can regulate signaling pathways responsible for cell death, division, differentiation, and migration (Assoian and Klein, 2008; Chen, 2008; Mammoto and Ingber, 2009; Nelson and Gleghorn, 2012; West-Foyle and Robinson, 2012). Changes in cellular and extra-cellular mechanical properties during malignant transformation potentially alter the forces acting on cells and thus influence morphogenetic evolution, proliferation, and invasion of cancer cells (Huang and Ingber, 2005; Lopez et al., 2008). It would be desirable to quantify this effect of mechanical properties on collective cell behavior in *in vivo* and *in vitro* multi-cellular systems. However, the presence of various other biological factors influencing cell behavior, as well as the complex interplay between the biological and mechanical factors makes it extremely difficult to isolate the effects of mechanical interactions. Computational modeling is an extremely useful tool in such conditions, where the effect of individual parameters can be studied and there is unlimited control on the parameter space.

Computational models can be developed to observe specific effects at various length scales ranging from the molecular to the macroscopic level, and these observations can then be integrated to obtain a complete picture of a specific process. Models have indeed been used extensively in understanding various aspects of cancer progression. Different models focusing on different aspects of cancer, such as effect of genetic heterogeneity, phenotypic evolution, biochemical interactions between cells and their surroundings, chemical and nutrient gradients, external forces, and mechanical interaction between cells, their neighbors, and the ECM can be found in literature. These models vary from being continuum based models of two evolving spatial domains representing the tumor mass and its environment to being discrete models where individual cells interacting with each other and the surroundings describe the system being simulated. A recent trend is to adopt a hybrid approach to incorporate the advantages of both continuum and discrete models into one, with a continuum description for the main tumor mass, and a discrete individual cell approach for tumor-environment interactions. The various modeling approaches have been reviewed in these references (Galle et al., 2006; Sanga et al., 2007; Byrne and Drasdo, 2009; Stolarska et al., 2009; Rejniak and McCawley, 2010; Deisboeck et al., 2011; Frieboes et al., 2011; Kam et al., 2012) and many others. Here we discuss some of these models, and a few other recent ones that examine the role of mechanics in tumor growth and invasion.

The goal of this review is to summarize the effects that changes in mechanical properties of cells and their surroundings have on tumor growth and metastasis as understood from computational models. There are a lot of models that incorporate some form of mechanical interaction between its elements, and many of them are progressions or off-shoots of previous models focusing more on the biochemical aspects of cancer progression. Hence, we shall focus only on key results regarding the influence of mechanical interactions rather than delve into the details of the model development process. With this information, we hope to display the importance of mechanistic models in identifying novel pathways of cancer progression, and direct the reader to more detailed sources on models interesting to them. Table 1 lists the specific changes in cellular and extra-cellular mechanical properties discussed here, experiments describing these changes and the corresponding observations on tumor cell behavior, as well as models that describe potential mechanisms connecting the two.

**Table 1:** **Some specific changes in cellular and extra-cellular mechanical properties, observations from experiments and computational models**.

Experimental observations	Model predictions
**Extra-cellular mechanical properties**	
Matrix stiffening (effect of increased density, cross-linking) (Paszek et al., 2005; Levental et al., 2009)	Increased cell proliferation driven by heterogeneity in ECM mechanical properties, protrusions along high density gradients (Macklin and Lowengrub, 2007; Rubenstein and Kaufman, 2008; Anderson et al., 2009; Macklin et al., 2009)
Matrix re-organization (effect of degradation and realignment) (Wolf et al., 2007; Friedl and Wolf, 2008)	Cell Proliferation driven by matrix degradation through the expression of MMPs and along realigned matrix fibers (Franks et al., 2005; Painter, 2009; Giverso et al., 2010; D’Antonio et al., 2013)
**Cellular mechanical properties**	
Increase in cell compliance or deformability (Cross et al., 2007; Fritsch et al., 2010; Jonas et al., 2011)	Tumorigenesis and increased malignancy, (Katira et al., 2012). Increased migration through porous ECM (Zaman, 2006; Zaman et al., 2007; Scianna and Preziosi, 2013)
Changes in cell adhesivity (Paredes et al., 2005; Ribeiro et al., 2010b)	Changes in tumor morphology, growth rates, and metastatic potential (Byrne and Chaplain, 1996; Armstrong et al., 2006; Ramis-Conde et al., 2008b; Frieboes et al., 2010; Rejniak et al., 2010; Katira et al., 2012)
Increase in cell contractility (Jonas et al., 2011; Kraning-Rush et al., 2012)	Increased migration rates and rigidity sensing (Moreo et al., 2008; Brodland and Veldhuis, 2012)

## Changes in Extra-Cellular Mechanical Properties

The extra-cellular environment of a carcinoma consists of surrounding healthy cells, a dense layer of fibrous basal membrane, and the surrounding stroma mainly comprised of fibrous matrix, adipocytes, and fibroblasts (Hogg et al., 1983). A growing tumor needs to push against this extra-cellular environment as it grows. Thus, intuitively, the stiffer the extra-cellular environment is, the less it deforms against the pressure applied by the growing tumor, restricting tumor size. This phenomenon was demonstrated experimentally by Helmlinger et al. (1997) and more recently by Cheng et al. (2009). Computationally this has been reproduced with varying levels of agreement by a variety of models (Chen et al., 2001; Ambrosi and Mollica, 2004; Drasdo and Hohme, 2005; Gevertz et al., 2008; Basan et al., 2009; Torquato, 2011; Montel et al., 2012; Ciarletta et al., 2013; Kim and Othmer, 2013) irrespective of model type (continuum, discrete, hybrid) and mechanism (growth retardation by formation of a necrotic core due to lack of nutrients, or by contact inhibition from increased packing density of growing cells, or both).

However, it is now known that the extra-cellular environment surrounding a tumor stiffens as the cells transform from normal to malignant to metastatic, and this transformation promotes cancer progression rather than arrests it (Paszek et al., 2005; Erler and Weaver, 2009; Klein et al., 2009; Levental et al., 2009; Ulrich et al., 2009). The models described above in their base form do not support this possibility. To explain the growth and metastasis of tumors against a dense, stiff, low porosity extra-cellular environment, models incorporating cell-ECM interactions are required. The continuum model described by Macklin and Lowengrub (2007) suggests that the aggressiveness of tumors growing in denser, stiffer environments that restrict cell mobility arises from increased shape instabilities during tumor growth and the formation of invasive finger-like morphologies. On the other hand, Franks et al. (2005) have suggested that tumor growth in a harsh environment like the one described above can lead to cell morphogenesis and progression toward a more malignant phenotype expressing high level of matrix degrading proteins (MMPs). These MMPs can then degrade the stiff, cross-linked ECM, weakening it. The growing tumor can then push against these weaker sections to grow as shown by D’Antonio et al. (2013). Chaplain et al. (2006) also incorporate a more active role of cell-ECM interactions in altering cell proliferation and migration rates to explain the growth of solid tumors against stiff extra-cellular environments. Their model focuses solely on mechanistic factors influencing tumor growth and incorporates the increase in ECM fiber density (Christensen, 1992; Kauppila et al., 1998; Brown et al., 1999) as well as changes in ECM degradation rates observed with malignant transformation (Clark et al., 2007; Alexander et al., 2008; Rizki et al., 2008). Increased ECM density facilitates cell proliferation as well as cell migration up to a certain extent (Zaman et al., 2006; Alexander et al., 2008). Increased MMP activity and corresponding degradation of the ECM also promotes cell migration through a dense ECM up to a certain extent (Erler and Weaver, 2009; Harjanto and Zaman, 2010). The effect of ECM density and cross-linking on cell invasiveness via the formation of invadopodia has been computationally modeled by Enderling et al. (2008). The effect of ECM degradation via the action of MMPs and resulting cell invasion has been modeled by Giverso et al. (2010). Based on the balance between the ECM fiber deposition and MMP degradation rates, as well as the spatial distribution of these factors in the tissue, various regimes of tumor growth, arrest, and invasion are possible. The heterogeneity arising in the tissue environment in terms of ECM density and stiffness because of these interactions can give rise to different morphologies for a growing tumor (Anderson et al., 2006; Frieboes et al., 2007; Gerlee and Anderson, 2008; Macklin and Lowengrub, 2008; Macklin et al., 2009; Trucu et al., 2013). Another model useful for studying the effect of ECM structure is described by Rubenstein and Kaufman (2008) where cell fate decisions are influenced by the neighboring elements and the overall interaction energy of the multi-cellular system. This allows for cell–cell as well as cell-ECM interactions to influence cell behavior and different collective phenomena can be observed based on the interaction rules. The model shows similar results as the described above, with increased cell proliferation near the densest ECM regions. Apart from cell proliferation and increased motility, changes in ECM structure can also impart directionality to the cells emanating from a growing tumor as shown computationally by Painter (2009). This is made possible through a mechanism know as contact guidance (Dunn and Heath, 1976; Guido and Tranquillo, 1993) where cells migrate along the length of ECM fibers. Thus, formation of aligned ECM fiber bundles can influence directed cell motility into the surrounding tissue.

## Changes in Cellular Mechanical Properties

Cells undergo very specific changes in their mechanical properties along with malignant transformation, just as the extra-cellular environment does. One particular change is the decrease in the stiffness of cells, or in other words, an increase in the compliance or deformability of cells. This has been observed for many different cancers. Furthermore, increased deformability of cells corresponds to higher malignancy and metastatic potential. This change in the mechanical property of cells complements that of the extra-cellular environment, which gets stiffer with increased malignancy. Using computational modeling Katira et al. (2012) have shown that decrease in cell stiffness can have a similar effect on cell proliferation rates as increase in the stiffness of the surroundings do (Klein et al., 2009). The model by Katira et al. incorporates the mechanical regulation of cell fate driven by changes in cell shape, and suggests that for cell clusters larger than a threshold size, the decrease in cell stiffness can drive uncontrolled growth and evasion of apoptosis in cells. While there are a number of other factors that influence cell proliferation, this seems to be a mechanistic pathway that aids tumor growth. The effect of changes in cell stiffness has also been studied by Drasdo and Hoehme (2012), where they look at the mechanical interactions between cells and a granular surrounding medium. Apart from tumor growth, the decrease in stiffness of cells has been shown to influence their ability to navigate tight turns during cell migration (Park et al., 2005; Lautenschlager et al., 2009). While the effect of this on cell migration during metastasis through the ECM is unknown, a potential increase in mobility can be predicted based on the models described in (Zaman et al., 2007; Scianna and Preziosi, 2013).

Apart from the lowering of cell stiffness, cells undergo changes in their binding ability with other cancer cells, normal cells, and the extra-cellular environment. These changes vary with cell phenotype and can be different at different stages of cancer progression. Also, their effects on tumor growth can vary based on the size and morphology of the tumor and the tumor-environment. For example studies have shown increased malignancy but non-invasiveness in tumors with increased P-cadherin binding between the cells (Van Marck et al., 2005). On the other hand studies have shown tumor growth arrest with increased E-cadherin binding. Other results have also shown increase in malignancy with decreased E-cadherin mediated adhesion (Bryan et al., 2008), while in still other cases the initiation of metastasis is driven by hypoxia induced loss of binding (Behrens et al., 1989; Finger and Giaccia, 2010). The effect of changes in cell adhesion has been studied in a lot of different modeling works (Drasdo and Hohme, 2005; Armstrong et al., 2006; Anderson et al., 2009; Bearer et al., 2009; Frieboes et al., 2010; Katira et al., 2012). One of the early models describing the effect of cell–cell adhesion on tumor growth is by Byrne and Chaplain (1996). The model balances the internal pressure of a growing tumor to the surface tension which is a function of the cell–cell adhesion. Thus changes in adhesion energies can drive instabilities in the contour profile of the growing tumor and result in finger-like extensions, representing metastasis. A model specifically suited for analyzing the effect of multiple changes occurring in the expression of cell-surface proteins that regulate cell–cell and cell-ECM interactions is the IBCell model described by Rejniak et al. (2010). The model describes cell behavior in terms of growth, phenotypic evolution, and apoptosis as a function of all the interactions it has with its neighbors and the different levels of surface proteins it is expressing at the time. This enables the prediction of a variety of different phenomena arising during malignant transformation and tumor growth. In principal this model is similar to the Rubenstein model mentioned previously, however the focused application described has been on the effect of changes in expressed cell-surface receptors and mechanical interactions between cells. Ramis-Conde et al. (2008a,b) describe a slightly different model focusing on the cadherin-catenin biochemical pathway and its effect on mechanical interaction between cells. The detachment of the cadherin bonds triggers the *wnt*-signaling pathway, and the model is able to predict epithelial to mesenchymal transition and cell migration toward a particular signal source.

## Future Directions

There are a number of models that describe the mechanics of cancer and the effect of specific changes in cellular and extra-cellular properties. However, it is necessary to combine these models focusing on different aspects of cell–cell and cell-ECM mechanical interactions into a unified theory of cancer progression. This comprehensive understanding of all the mechanical aspects is required in order to predict clinically observed tumor growth and metastasis, and decouple the mechanics from the biology. The idea that a select few changes in cellular and extra-cellular mechanical properties can promote the growth of a malignant phenotype of cancer is intriguing. As depicted in Figure 1, there is a strong interplay between biological and mechanical factors involved in cancer progression, with each one influencing the other. This opens up the possibility of mechanical regulation and manipulation of cell behavior to alter cancer outcome. Researchers can develop tools to predict and treat cancer that are focused on rectifying the few mechanical property changes (for examples refer to Lekka et al., 2001; Cross et al., 2011) as compared to vast number of heterogeneous genetic and epigenetic factors associated with cancer progression (Swanton et al., 2011; Visvader, 2011).

## Conflict of Interest Statement

The authors declare that the research was conducted in the absence of any commercial or financial relationships that could be construed as a potential conflict of interest.
